# Growing ZnO Nanoparticles on Polydopamine-Templated Cotton Fabrics for Durable Antimicrobial Activity and UV Protection

**DOI:** 10.3390/polym10050495

**Published:** 2018-05-03

**Authors:** Jianhua Ran, Mantang He, Wenbin Li, Deshan Cheng, Xin Wang

**Affiliations:** 1 School of Textile Science and Engineering, Wuhan Textile University, Wuhan 430200, China; jhran@wtu.edu.cn (J.R.); mantang_he.wtu@yahoo.com (M.H.); li780713@126.com (W.L.); 2 State Key Laboratory for Hubei New Textile Materials and Advanced Processing Technology, Wuhan 430200, China; 3 School of Fashion and Textiles, RMIT University, Melbourne, VIC 3056, Australia

**Keywords:** ZnO nanoparticles, polydopamine, cotton fabrics, antimicrobial, UV protection

## Abstract

This work aims to develop durable functional cotton fabrics by growing zinc oxide (ZnO) nanoparticles on polydopamine (PDA) templates. ZnO nanoparticles were grown on the PDA-templated cotton fabrics by the hydrothermal method at room temperature. The surface morphology, chemical composition, and crystalline structure of the ZnO-coated cotton fabrics were characterized by scanning electron microscope (SEM) with energy dispersive X-ray analysis (EDX), X-ray diffraction (XRD), and X-ray photoelectron spectroscopy (XPS). The ZnO nanoparticles were found to disperse evenly on the surface of cotton fabrics. The ultraviolet (UV) protection factor (UPF) value of the ZnO-coated cotton fabrics was maintained at 122.5, and 99% reduction in bacterial load was observed against *Gluconobacter cerinus* even after five cycles of laundering. The PDA was found to be effective in fixing the ZnO seeds tightly on the surface of cotton fabrics, resulting in excellent durability of the coating of ZnO nanoparticles.

## 1. Introduction

Functionalization of cotton fabrics is necessary in developing protective clothing and performance textiles [1]. There are many methods by which to functionalize the surface of cotton fabrics, such as coating [2], plasma treatment [3], surface modification [4], grafting [5], and growing of nanoparticles [6]. The most common method is impregnating nanoparticles on the surface of cotton fabrics to endow special properties. Zinc oxide (ZnO) is one of the most applied metallic oxides with high stability, nontoxicity, wide-band-gap semiconductor properties, and low-cost synthesis. It has been applied to endow new functions on textiles, such as antimicrobial activity [7], UV protection [8], self-cleaning [9], and superhydrophobicity [10]. Several methods have been developed to immobilize nanoparticles on the surface of cotton fabrics, including sol–gel [11], pulsed laser deposition [12], ultrasound irradiation [13], self-assembly [14], electroless deposition [15], and hydrothermal methods [16,17,18]. Among these methods, the hydrothermal method is widely used because it is simple and no special equipment is required, and the good dispersion of nanoparticles can be expected. The resulting nanoparticles from this method have good growth orientation with the morphology controllable by varying the hydrothermal conditions. However, seeding of ZnO on cotton fabrics requires the pad-dry-cure method, in which high temperature is usually applied [19]. The original properties of cotton fabrics will be deteriorated after high temperature treatment and the treatment itself is not cost effective. Thus, novel hydrothermal methods are needed to further commercialize this technique in industry. 

Biomimetic modification of existing polymers to develop biopolymers and nanocomposites has attracted research attention [20,21,22]. Inspired by nature, dopamine has been discovered to be suitable for forming polydopamine (PDA) layers on almost any surface, with the potential in creating versatile functional coatings [23]. PDA is rich in catechol and amine functional groups to ensure strong adhesion to any substrate, resulting in a durable and strong coated surface on substrates [24]. The formed PDA layer contains reactive groups to act as a versatile secondary functional platform. PDA has recently been used as a reducing agent for reducing metal ions to immobilize nanoparticles on textiles, and certain studies mainly focused on depositing nanoparticles including silver, silica, copper oxide, ferriferrous oxide, and titanium dioxide on the surface of fibers and fabrics by in situ synthesis [25,26,27,28,29].

In this work, a facile method was developed by which to grow ZnO nanoparticles on PDA templates on the surface of cotton fabrics. The PDA layer was firstly adhered onto the surface of cotton fabric via oxidative polymerization of dopamine. The PDA-templated cotton fabrics were immersed in a sol of ZnO seeds, followed by the growth of ZnO nanoparticles from zinc precursors in an aqueous medium. The surface morphology and chemical structure together with functions including antimicrobial activity and UV protection of the ZnO-coated cotton fabrics were characterized and investigated for further application of this research.

## 2. Experimental

### 2.1. Materials

Cotton fabrics (plain, 135 g/m^2^ mass, density 570/10 cm warp and 280/10 cm weft) were obtained from Yudahua Textile Co., Ltd., Wuhan, China. 3-Hydroxytyramine hydrochloride (dopamine hydrochloride) was purchased from Aldrich Chemical Co., Milwaukee, WI, USA. Zinc acetate dihydrate (Zn(CH_3_COO)_2_·2H_2_O, 98%), triethylamine (C_6_H_15_N, 99%), isopropyl alcohol (C_3_H_8_O, 99%), sodium hydroxide (NaOH, 97%), zinc nitrate hexahydrate (Zn(NO_3_)_2_·6H_2_O, 98%), and hexamethylenetetramine (C_6_H_12_N_4_, 99%) were obtained from Aladdin Chemical Agent Co., Shanghai, China. All the chemicals were of analytical reagent grade and used without further purification.

### 2.2. Preparation of ZnO-Coated Cotton Fabrics

**Figure 1:**
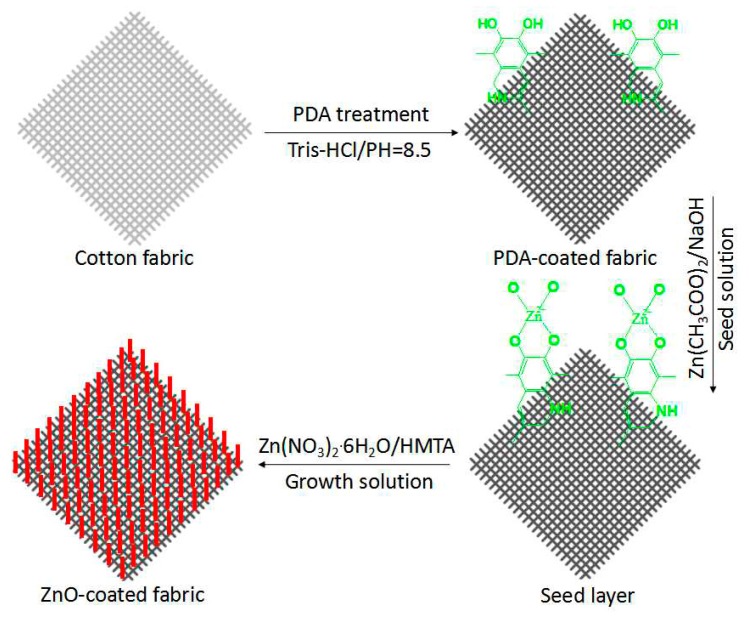
Schematics of the growth of ZnO nanoparticles on polydopamine (PDA)-templated cotton fabrics.

A hydrothermal method was used to grow ZnO nanoparticles on PDA-templated cotton fabrics, as illustrated in Figure 1. Cotton fabrics were cleaned by acetone and then by deionized water. Dopamine solution (10 mM) was dissolved in a Tris buffer solution with the pH value of 8.5 by adding HCl. Cotton fabrics were dipped into the dopamine solution at room temperature with stirring for 24 h. The fabrics were then rinsed with distilled water and dried in a vacuum oven.

The ZnO seed solution was prepared by adding 0.02 M zinc acetate into methanol under vigorous stirring at 60 °C, followed by a dropwise injection of 0.03 M NaOH in methanol into the solution. After the solution was continuously stirred and sustained at 60 °C for 2 h, the solution became clear. The PDA-templated cotton fabrics were dipped into the mixed solution for 20 min, pressed against the smooth rubber roll, and dried in a vacuum oven.

The ZnO growth solution was obtained by mixing equimolar zinc nitrate hexahydrate (Zn(NO_3_)_2_·6H_2_O) and hexamethylenetetramine (C_6_H_12_N_4_, HMTA) aqueous solutions at room temperature. The seeded cotton fabrics were then immersed into the solution in a water bath of 90 °C for 5 h. The ZnO-coated cotton fabrics were then rinsed with distilled water and dried. 

### 2.3. Characterizations and Measurements

The surface morphology and elemental composition of cotton fabrics were observed on a scanning electron microscopy (SEM, JSM-5600LV, JEOL, Tokyo, Japan) with an energy dispersive X-ray spectrum (EDX, Oxford Instruments, Oxford, UK). 

XPS measurements were performed on a PHI 5000C ESCA system with a Mg Kα source at 14.0 kV and 25 mA (Perkin-Elmer, American Fork, UT, USA). 

The X-ray diffraction (XRD) spectra were collected on an X-ray diffractometer (D/max 2500, Rigaku, Tokyo, Japan) using Cu Kα radiation with the diffraction angle range 2θ = 10–80°, at 40 kV and 200 mA. 

Ultraviolet–visible spectroscopy (UV–Vis) (U-4100, Hitachi, Tokyo, Japan) was used to characterize the cotton fabric samples. 

Thermogravimetric analysis (TGA) was conducted on a Netzsch TG209 F1 thermal analyzer (Burlington, MA, USA), with a heating rate of 10 °C/min from 30 to 700 °C in a nitrogen atmosphere at a flow rate of 60 mL/min. The instrument calibration before experiments included balance correction and temperature correction, according to the methods offered by the instrument manufacturer. The temperature was reproducible to ±0.1 °C and the mass to ±0.1%.

The ultraviolet protection factor (UPF) of the coated cotton samples was measured using a UV-1000F ultraviolet transmission analyzer (North Sutton, NH, USA) according to the standard GB/T 18830-2002. The durability of the coated cotton samples was assessed by the UPF of fabric after washing in a Haier automatic washing machine (Qingdao, China) according to the AATCC Test Method 135-2000. 

Antimicrobial activity was tested using *Gluconobacter cerinus*. The bacteria were cultivated at 37 °C in a yeast–dextrose broth containing 10 g/L peptone, 5 g/L sodium chloride, and 5 g/L yeast extract. The bacteria were diluted with a 0.1 mol/L phosphate buffer solution (PBS, pH = 7.0) to a certain concentration. The bacterial suspension was centrifuged at 3000 rpm for 10 min for the antibacterial assay. After the supernatant being removed, the cells were washed for three times with PBS and resuspended in PBS with a concentration of 10^8^ cells/mL in a sterile Erlenmeyer flask. The flask was then subjected to shaking at 200 rpm at 37 °C for 2 h. The samples were then transferred to Petri dishes, and solid growth agar was added immediately. The number of viable colonies was counted manually after incubation of the plates at 37 °C for 24 h. The bactericidal efficiency (*Y*) was calculated according to the following formula:

(1) $$Y=\frac{W_{c}-W_{s}}{W_{c}}\times 100\%$$

where *W*_c_ is the average number of the colonies on the pristine cotton fabric and *W*_s_ is the average number of the colonies on the coated cotton samples.

## 3. Results and Discussion

### 3.1. Mechanism

The self-polymerization of dopamine has brought active groups including –OH and –NH_2_ to the surface of the cotton fabrics. Once the PDA-coated cotton fabrics were immersed into the Zn(CH_3_COO)_2_ and NaOH mixed solution, these active groups reacted with ZnO seeds to fix them on the surface of the PDA template [30]. The associated reactions of the preparation of the ZnO seeds are shown in Equations (2) and (3).

Zn(CH_3_COO)_2_ + 2NaOH → Zn(OH)_2_↓ + 2CH_3_COONa (2)

(3) $$\mathrm{Zn}(\mathrm{OH}{)}_{2}\overset{\mathrm{heat}}{\to }\mathrm{ZnO}+H_{2}O$$

The seeded fabrics were then immersed into the mixed solution of Zn(NO_3_)_2_·6H_2_O and HMTA. The HMTA was hydrolyzed in aqueous solution to generate OH^−^ ions, and the OH^−^ ions complexed with Zn^2+^ ions to form Zn(OH)_2_. The reaction suspension containing the Zn(OH)_2_ particles was further heated to obtain ZnO nanoparticles [31]. The associated reactions of the growth of ZnO nanoparticles on the seeded fabrics are shown in Equations (4) and (5).

C_6_H_12_N_4_ + 6H_2_O → 6H_2_CO + 4NH_3_ → NH4^+^ + OH^−^ (4)

(5) $${\mathrm{Zn}}^{2+}+2{\mathrm{OH}}^{-}\to \mathrm{Zn}(\mathrm{OH}{)}_{2}↓\;\overset{\mathrm{heat}}{\to }\mathrm{ZnO}$$

### 3.2. Characterizations

#### 3.2.1. Morphology

**Figure 2:**
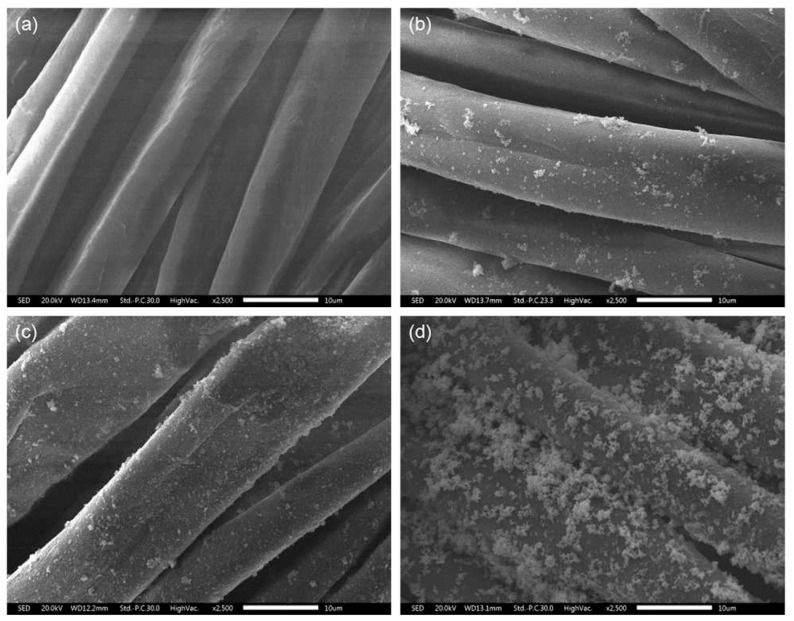
Surface morphology of pristine cotton fabrics (**a**), PDA-templated cotton fabrics (**b**), seeded cotton fabrics (**c**), and ZnO-coated cotton fabrics (**d**). Bar = 10 μm.

The growing of ZnO nanoparticles on the surface of cotton fabrics has completely changed the surface morphology of the fibers, as shown by the SEM photos in Figure 2. The pristine cotton fibers are smooth with longitudinal convolutions (Figure 2a), whereas the surface of PDA-templated fibers are covered by a film with some particles due to the polymerization of dopamine (Figure 2b). Furthermore, ZnO nanocrystals have formed discrete nucleation sites uniformly on the surface of the cotton fibers after the seeding process (Figure 2c). The ZnO nanocrystals on the surface of cotton fibers are due to the reaction of the active functional groups of the polydopamine and ZnO crystals. The nucleation step is crucial for the further growth of ZnO nanoparticles. As a result, the surface of the cotton fibers is fully covered with a layer of ZnO nanoparticles (Figure 2d). Most of the nanoparticles have a rod shape with an approximate length of 1.76 ± 0.12 μm. 

#### 3.2.2. EDX Analysis

**Figure 3:**
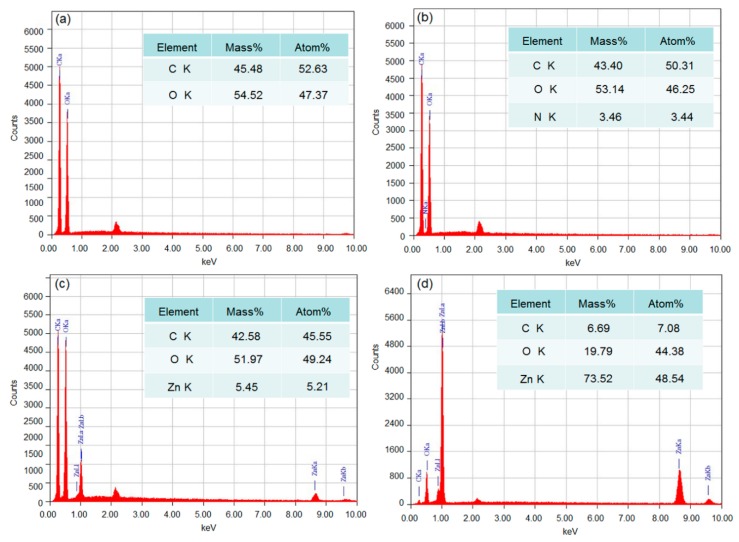
EDX spectra of pristine cotton fabrics (**a**), PDA-templated cotton fabrics (**b**), seeded cotton fabrics (**c**), and ZnO-coated cotton fabrics (**d**).

EDX analysis was used to determine the surface elements together with their percentages of the coated cotton fabrics. The EDX spectra of cotton fabrics before and after coating with ZnO nanoparticles are displayed in Figure 3. For the pristine cotton fabrics, only two peaks corresponding to carbon (C) and oxygen (O) elements are observed in Figure 3a. The EDX spectrum of PDA-templated cotton fabrics presents the nitrogen element (Figure 3b) with a weight percentage of 5.46%. The nitrogen element originates from dopamine due to the successful polymerization of dopamine on the fabrics. Figure 3c shows the EDX spectrum of the seeded fabrics, and the Zn element can be found with a weight percentage of 5.21%. The EDX spectrum of ZnO-coated cotton fabrics in Figure 3d shows a Zn content of 73.52%, indicating that the fiber surface is almost completely covered with ZnO. The detected Zn element on the surface is consistent with the observation of the surface morphology in Figure 2d. 

#### 3.2.3. XPS Analysis

**Figure 4:**
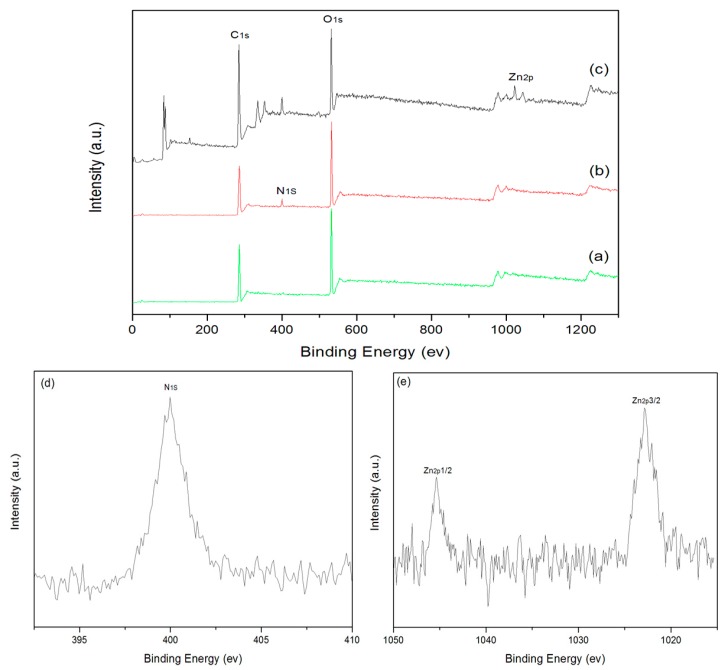
XPS spectra of pristine cotton fabrics (**a**), PDA-templated fabrics (**b**), and ZnO-coated fabrics (**c**). High-resolution scan of the N1s peak (**d**) and Zn2p peak (**e**).

The chemical state of elements on the cotton fabrics were monitored by XPS in the process of coating, as shown by the wide-scan and core-level XPS spectra in Figure 4. The XPS spectrum of the pristine cotton fabrics (Figure 4a) indicates two characteristic peaks corresponding to C1s and O1s, and that of PDA-templated cotton fabrics (Figure 4b) shows an extra characteristic peak corresponding to N1s. The percentage of the nitrogen content is 7.53%, which is in accordance with the EDX results. The high-resolution scan of N1s (Figure 4d) shows a peak at 400 eV, and the peak is attributed to the amino groups of dopamine [32]. These results further reveal that the surface of cotton fabrics has been successfully templated with polydopamine. An extra Zn2p peak can be detected for the ZnO-coated cotton fabrics (Figure 4c), indicating the presence of ZnO nanoparticles on the surface of cotton fabrics. As shown by the high-resolution scan in Figure 4e, there are two peaks with binding energy values of 1022.7 and 1045.3 eV, corresponding to Zn 2p3/2 and Zn 2p1/2, respectively. These values are identical to that of ZnO crystals [33]. The XPS results confirm that ZnO nanoparticles have been grown properly on the surface of the PDA templates. 

#### 3.2.4. Crystalline Structure

**Figure 5:**
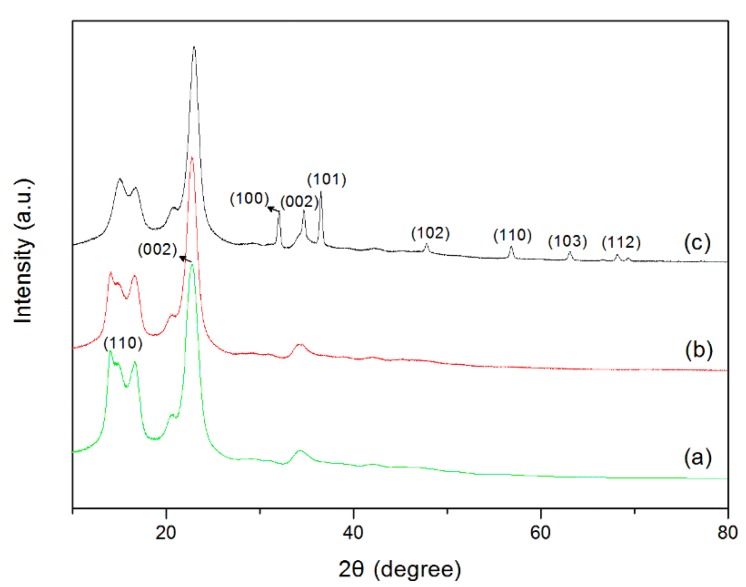
XRD patterns of pristine cotton fabrics (**a**), PDA-templated fabrics (**b**), and ZnO-coated fabrics (**c**).

The XRD pattern of the pristine cotton fabrics (Figure 5a) exhibits diffraction peaks at 2θ = 14.9°, 16.6°, and 22.7°, corresponding to (1–10), (110), and (002) planes, respectively. These are the diffraction peaks of the type I cellulose characteristics (JCPDS No. 03-0226) [34]. A similar XRD pattern can be observed in Figure 5b for the dopamine-coated cotton fabrics, suggesting that PDA templating has little influence on the crystalline structure of cotton fabrics. However, the XRD pattern of the ZnO-coated cotton fabrics exhibits extra diffraction peaks at 2θ = 32.1°, 34.7°, 36.5°, 47.8°, 56.7°, 63.1°, and 68.1°. These peaks are attributed to the (100), (002), (101), (102), (110), (103), and (112) planes of hexagonal ZnO particles, matching well with the Joint Committee on the Powder Diffraction Standard (JCPDS No. 36-1451) [35]. These results further prove that ZnO nanoparticles have been grown on the surface of the cotton fabrics.

#### 3.2.5. UV–Vis Spectrophotometry

**Figure 6:**
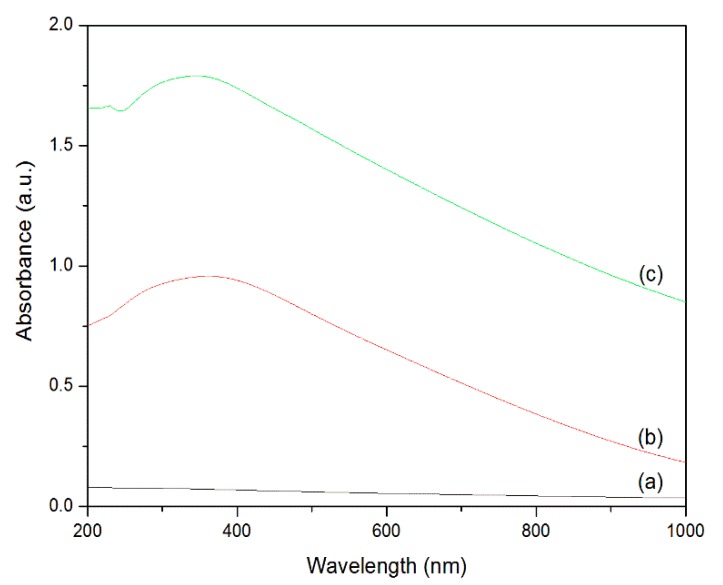
UV–vis spectra of pristine cotton fabrics (**a**), PDA-templated fabrics (**b**), and ZnO-coated fabrics (**c**).

The optical properties of the cotton fabrics were assessed by UV–Vis transmission spectroscopy in the process of coating. Figure 6a depicts the absorbance spectra of the pristine cotton fabrics, and it does not exhibit any absorption peaks within the spectral region. The spectrum of the PDA-templated cotton fabrics (Figure 6b) has a broad absorption band. During the self-polymerization, the catechol groups of dopamine were oxidized with a large amount of melanin generated [36]. The generated melanin absorbs ultraviolet and visible lights in a wide range. The absorbance spectrum of the ZnO-coated cotton fabrics (Figure 6c) presents an absorption band at 359 nm, which is attributed to the strong UV absorbance of ZnO nanoparticles. The results suggest that the ZnO-coated cotton fabrics are very effective at absorbing ultraviolet light.

#### 3.2.6. Thermogravimetric Analysis 

**Figure 7:**
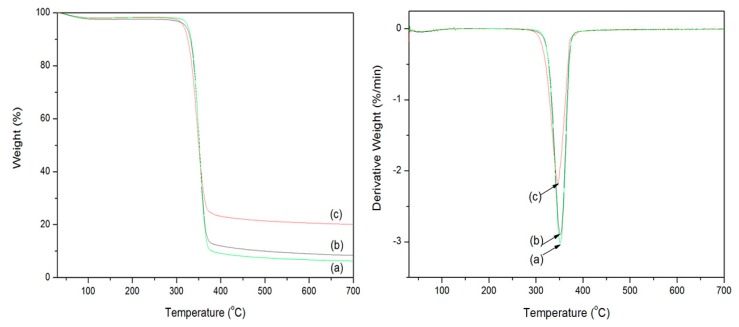
TG and DTG curves of pristine cotton fabrics (**a**), PDA-templated fabrics (**b**), and ZnO-coated fabrics (**c**).

Thermogravimetric analysis (TGA) was employed to measure the thermal stability of the cotton fabrics before and after coating under an N_2_ atmosphere. As shown in Figure 7a, the weight loss of the pristine cotton fabrics is 5% at 326 °C, and the thermal decomposition of cellulose cleavage mainly occurs in the range of 326–375 °C. The maximum weight loss appears at 352 °C, with only 6.25% residue left at the end of the test (700 °C). The DTG curve also reveals that the temperature of the maximum degradation rate is 352 °C. The PDA-templated cotton fabrics (Figure 7b) show a weight loss of 5% at 321 °C, and the maximum mass loss occurs at about 351 °C, with 8.42% char remaining in the end. The temperature of the maximum degradation rate decreased to 351 °C after the coating of PDA. The PDA-templated cotton fabrics show a slightly lower initial temperature and maximum-rate degradation temperature than that of the pristine cotton fabrics, and this is attributed to the earlier decomposition of PDA. The deposited products on the fiber surface due to the decomposition of PDA have a small effect on the thermal stability of the cotton fabrics [29]. The ZnO-coated cotton fabrics (Figure 7c) indicate a weight loss of 5% at 346 °C, and the maximum mass loss occurs at about 345 °C, with 20.14% residue in the end. The temperature of the maximum degradation rate decreased to 346 °C after the coating of ZnO nanoparticles. The increased residue of coated cotton fabrics may be ascribed to the immobilized PDA and ZnO nanoparticles. The maximum-rate degradation temperature of the coated cotton samples is lower than that of the pristine cotton fabrics. The changed thermal degradation performance of the coated cotton fabrics is ascribed to the damage of the cotton fibers by sodium hydroxide in the coating process. The TG results indicate that the thermal stability of the cotton fabrics has not been substantially affected by the coating of ZnO nanoparticles.

### 3.3. Functions

#### 3.3.1. UV Protection

In order to investigate the UV-protective properties of the ZnO-coated cotton fabrics, the UPF value and transmittance of UVA (320–400 nm) and UVB radiation (290–320 nm) were measured with results shown in Table 1. The UPF value of the pristine cotton fabrics is as low as 6.4, with the UVA and UVB transmittance values of 10.68% and 8.45%, respectively. It is evident that light can penetrate through cotton fabrics and their protection against UV radiation is very poor. The UPF value of the PDA-templated cotton fabrics is 36.8, with the UVA and UVB transmittance values of 2.1% and 1.6%, respectively. The enhanced UV protection is due to the large amount of melanin on the surface of cotton fibers as a result of the self-polymerization of dopamine. The melanin can absorb ultraviolet light, and thus the UV-protection capacity of the templated cotton fabrics is enhanced. The UPF value of the ZnO-coated cotton fabrics is 157.8, with the UVA and UVB transmittance values of 0.46% and 0.31%, respectively, whereas the UPF of the ZnO-coated cotton fabrics by a conventional coating method is 65.93 [37]. It is evident that the coating of ZnO nanoparticles has endowed excellent UV protection to the cotton fabrics, and this is due to the intrinsic high UV absorbance and scattering properties of the ZnO nanoparticles. 

**Figure 8:**
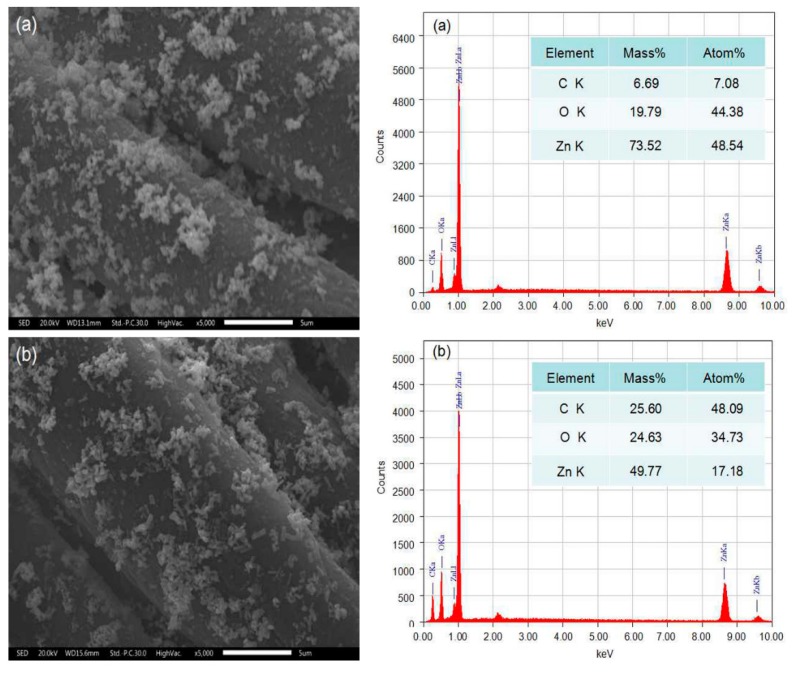
Surface morphology with EDX spectra of ZnO-coated fabrics before (**a**) and after (**b**) five washing cycles. Bar = 5 μm.

After five washing cycles, the UPF value of the ZnO-coated cotton fabrics was maintained at 122.5, suggesting a good durability of the coated ZnO nanoparticles against washing. Some of the uncapped ZnO nanoparticles on cotton fabrics can be easily washed out in the laundering process, leading to the decrease of the UPF value. The excellent durability can be attributed to enhanced binding efficiency of the ZnO nanoparticles on the PDA-templated cotton fabrics. The ZnO-coating by the conventional coating method shows extremely poor durability, with a severe drop in UPF after ten washing cycles [37,38]. The PDA templates have stabilized ZnO nanoparticles on the surface, thus the UPF value after five washing cycles is much higher than ZnO-coated fabrics made by the conventional method [38]. Surface morphology observation indicates that ZnO nanoparticles are immobilized on the surface of fibers after five washing cycles, as shown in Figure 8. EDX spectra show that the weight percentage of Zn has decreased from 73.52% to 49.77% after washing. 

#### 3.3.2. Antibacterial Activity

**Figure 9:**
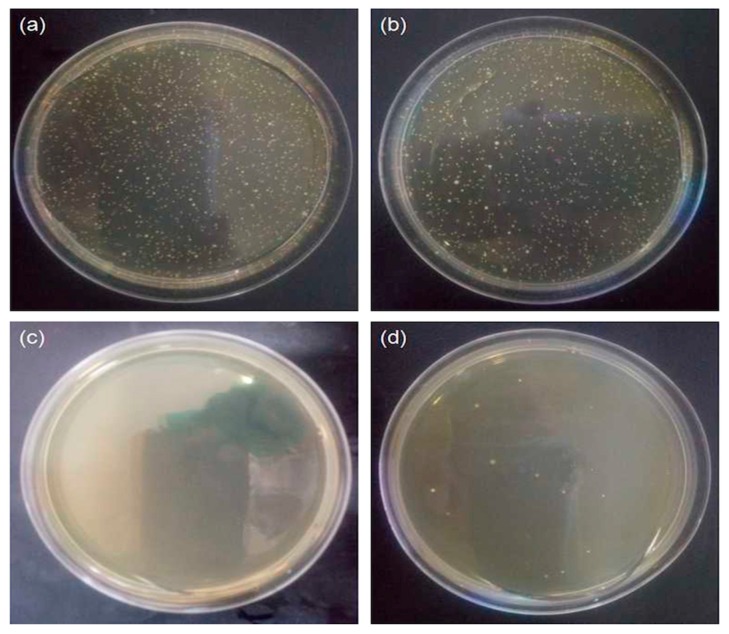
Antimicrobial activity of pristine cotton fabrics (**a**), PDA-templated cotton fabrics (**b**), ZnO-coated cotton fabrics (**c**), and ZnO-coated cotton fabrics after five washing cycles (**d**).

The antimicrobial activity of the pristine cotton fabrics and ZnO-coated cotton fabrics were evaluated using a colony count method, as shown in Figure 9. The petri dish is covered with bacteria, which reveals that the pristine cotton fabrics do not have any antibacterial properties (Figure 9a). As shown in Figure 9b, with the result of the PDA-templated cotton fabrics, the bacteria have not grown satisfactorily on the petri dish. The result suggests that the antimicrobial activity of the PDA-templated cotton fabrics has been enhanced. The antimicrobial assay of the ZnO-coated cotton fabrics (Figure 9c) indicates a 100% reduction in bacterial load against *Gluconobacter cerinus*. ZnO nanoparticle-coated cotton fabrics made by the conventional method have shown a reduction percentage of 96.3% against bacteria [39], whereas the reduction percentage from this work is 100%. The excellent antimicrobial activity of the coated cotton fabrics can be attributed to the coated ZnO nanoparticles producing reactive oxygen species and perishing the microbial membrane [40]. After five washing cycles, the antimicrobial assaying of the ZnO-coated cotton fabrics (Figure 9d) shows that the bacteria have not grown, and a 99% reduction in bacterial load was observed against *Gluconobacter cerinus*. The strong adhesive ability of PDA results in the durable coating of ZnO nanoparticles on cotton fabrics, and thus the excellent antibacterial properties have been preserved after washing.

## 4. Conclusions

ZnO nanoparticles were successfully grown on the polydopamine (PDA)-templated cotton fabrics by the hydrothermal method. The SEM photos showed that the ZnO nanoparticles are well dispersed on the surface of the fibers, with a rod shape and uniform size. The surface composition and crystallinity structure analysis of the ZnO-coated cotton fabrics by XPS, EDX, and XRD confirmed the presence of ZnO nanoparticles on the cotton fabrics. The TG results indicated that the coating of ZnO nanoparticles has little effect on the thermal stability of the fabrics. The UPF value of the ZnO-coated cotton fabrics was 157.8, and the value was maintained at 122.5 even after five washing cycles. The ZnO-coated cotton fabrics exhibit very good antibacterial properties against *Gluconobacter cerinus*, and the antimicrobial activity was preserved properly after the fabrics were washed for five cycles. The PDA template has immobilized the ZnO nanoparticles firmly on the surface of cotton fabrics, bringing excellent durability to the coated cotton fabrics.

## Figures and Tables

**Table 1:** UPF values, transmission (T) in the UVA and UVB regions, and UPF rating of the cotton fabrics.

Samples	UPF	T (UVA) (%)	T (UVB) (%)	UPF Rating
Pristine cotton fabrics	6.4	10.68	8.45	poor
PDA-templated cotton fabrics	36.8	2.1	1.6	good
ZnO-coated cotton fabrics	157.8	0.46	0.31	excellent
